# Platelet Septic Transfusion Reactions in Patients With Hemato-Oncological Diseases

**Published:** 2017-04-01

**Authors:** Farhad Razjou, Abolfazl Dabir Moghaddam, Gharib Karimi, Maryam Zadsar

**Affiliations:** *Blood Transfusion Research Centre, High institute for Research and Education in transfusion Medicine. Tehran, Iran*

**Keywords:** Septic Transfusion Reaction, *Citrobacterfreundii*, Patients With Leukemia or Lymphoma, Platelet Concentrates, Hemovigilance

## Abstract

**Background & Objective:**

Bacterial, contamination of blood components are a significant risk for transfusion reactions. Inherently, platelet concentrates (PCs) are vulnerable to bacterial contamination, due to the storage condition of processed PCs at room temperature, which provide very suitable conditions for the proliferation of microorganisms.

The current study aimed at investigating the transfusion associated septic reaction rate in patients with hemato-oncological diseases in Imam Khomeini Hospital, Tehran, Iran, and identifying the contaminating bacteria.

**Methods:**

A total of 3056 adult patients of the Cancer Center of Imam Khomeini Hospital in Tehran transfused with PCs were studied based on the clinical symptoms of septic transfusion reaction from June 1, 2010 to May 31, 2011. Patient presented with the criteria of reaction and the residual components were evaluated for bacterial contamination by Bac T/Alert system.

**Results:**

Patients with leukemia or lymphoma transfused with random-donor PCs were evaluated the signs and symptoms of transfusion reaction occurred only in 12 (%0.4) cases. Automated cultivation found 3 positive blood cultures. Among these a male recipient was categorized as possible septic transfusion reaction and *Citrobacterfreundii* was isolated from blood sample.

**Conclusion:**

Appropriate clinical utilization of PCs transfusion, and ongoing vigilance to recognize, investigate, promptly treat, and report all suspicious transfusion reactions are necessary to manage the transfusion complication including transfusion-transmitted infections (TTI).

## Introduction

Platelets (PLT) transfusion is practiced as one of the most important therapeutic or prophylactic interventions on patients with hemato-oncological diseases. Each year about 10 million PLT concentrates (PCs) are transfused in the United States (1). In Tehran province, Iran, almost 241 000 PCs are distributed annually, but data on the rate of transfusion are unavailable. In the United States of America, almost 17% of transfusion related mortalities are caused by bacterial contamination and septic reactions after allogeneic transfusion of PCs (2).

Accomplishment of improved blood donor selection standards and skin disinfection prior to donation, first-aliquot diversion, PCs bacterial culture as routine quality control process, and somehow automated cultural screening significantly reduced the risk of transfusion reactions associated with bacterially contaminated blood components in the last decade, but still there is a significant risk (3). According to the American Association of Blood Banking (AABB) standards 2012, (3) there are different methods to detect bacterial contamination of PCs by the Blood Collection Centers (BCCs) in the developed countries.

Inherently, PCs are vulnerable to bacterial contamination due to the storage condition of PCs at room temperature, which provides very suitable conditions for microorganisms’ proliferation (4).

Transient asymptomatic bacteremia of blood donors during blood donation, donor skin microflora, environmental contamination during the process, and storage period are the main routs of infection (4). 

Pre transfusion bacteriological screening for PCs was implemented in Europe through the late 90's and in North America from March 2004. Currently, European BCCs are forced to use either Bac T/Alert system with 1-10 CFU/mL sensitivity and >90% specificity, or Pall eBDS system with respectively, 1-5 CFU/mL sensitivity and >90% specificity to screen PC products and release the negative products to be distributed, but cultivation should be continued till the 7th day and if further microorganism growth detected the products should be either recalled, or passed into the look back process (3). Sometimes other rapid tests are implemented immediately before transfusion in the Europe and the US; such as visual examination by detection limit of 10^7^-10^8^ CFU/mL, PGD, Bac-Detect, BactiFlow, and Milliflex by detection limit of >10^3^ and 90% specificity and ThromboLUX, BactTx, and Nucleic acid-based methods with >10^3^sensitivity and >90% specificity (5).

Septic reaction due to PCs' transfusion is presented by clinical symptoms such as fever, shaking chills, tachycardia, hypotension, disseminated intravascular coagulation (DIC), rash, generalized pain, multiorgan failure, and finally shock and death (6). Furthermore, the aforementioned clinical symptoms occur not necessarily due to septic reactions; it could be caused by other diseases or noninfectious transfusion adverse reactions such as transfusion related acute lung injury (TRALI), allergic/anaphylactic reactions, hemolytic transfusion, and nosocomial infections (7).

The clinical symptoms may be presented during or after transfusion until 24 hours later or even delayed for much extended time (8). The severity of septic reaction depends on species and violence of bacteria, the loading dose, clinical condition, and background disease of the recipient (8, 9).

In Iranian Blood Banking Organization (IBTO), 1% of total amount of daily products are cultured to evaluate the risk of transfusion-transmitted bacterial infection (TTBI) and monitor good manufacturing practice (GMP) application to produce and process the blood products. As mentioned above, no screening method is implemented for bacterial threats yet. 

The current study aimed at investigating the transfusion-transmitted bacterial reaction rate in patients with hemato-oncological diseases admitted into Imam Khomeini Hospital, Tehran, and identifying the contaminating bacteria.

## Material and Methods

A total of 3056 adult patients with lymphoma or leukemia, the Cancer Center of Imam Khomeini Hospital were enrolled into the study from June 1, 2010 to May 31, 2011. They were studied based on the symptoms of septic transfusion reaction by the previously described methods (10, 11). A trained physician and nurse reviewed the patients’ conditions; each patient who presented the symptoms of reaction signed the informed consent to be tested by Bac T/Alert automated culture. A 10-mL venous blood was taken from each patient to detect aerobic and anaerobic bacteria by employing Bac T/Alert standard bottles named bacterial platelet aerobic (BPA) and bacterial platelet anaerobic (BPN) (Biomerieux, Inc., Durham, NC), respectively (9).

In addition, bacterial contamination of the residual component was evaluated by culturing the residue of transfused products employing Bac T/Alert standard bottles named BPA and BPN (Biomerieux, Inc., Durham, NC) to detect aerobic and anaerobic bacteria, respectively.

If the content of PC residue was not enough, 40 to 50 mL of tryptic soy broth was added as a culture broth to the residual content under sterile condition and was completely mixed; thereafter, about 20 mL of mixture was drown and 10 mL was inoculated into each aerobic and anaerobic bottle. The bottles were incubated for 14 days at 36^o^C to enhance detection of slow growing microorganisms and obligatory anaerobes. If a bottle was detected positive, then it was confirmed by culture-based bacterial detection method. 

Isolated bacteria were identified by Mini Api (Biomerieux, Inc., Durham, NC) apparatus and standard Rapid ID 32E (Biomerieux, Inc., Durham, NC) test kits.

The inclusion criteria were as follows: symptoms beginning within 4 hours of platelet transfusion including fever ≥39^°^C, or increase in body temperature (>2^°^C) from pretransfusion point, rigors, tachycardia which is defined as pulse rate ≥120 beat per minute (bmp), or increase in pulse rate (>40 bmp) from pretransfusion value. In addition, the variation in blood pressure ≥30 mmHg was also precisely observed and if detected, further investigation was recommended. 

The patients with transfusion of other blood components were excluded from the study.

Each symptomatic patient was investigated for the other causes of transfusion reactions eg; acute hemolytic transfusion reaction (AHTR), febrile non-hemolytic transfusion reaction (FNHTR), allergic and anaphylactic reactions. 

Septic transfusion reactions were classified into 3 groups: definite, probable, and possible (10).

A definite septic transfusion reaction was diagnosed with the presence of the relevant clinical symptoms and similarity between the results of patient blood culture and the cultivated inclusive residue of blood component sample (Table 1).

**Table 1:** Classification of Bacterial Contamination in Platelet Concentrates Based on BACTHEM Hemovigilance System

patients\Tests	Signs and Symptoms	Blood Culture Results	The Residual Culture
Definite*	+	+	+
Probable	+	+ / -	+
Possible	+	+	/or not done

* The bacterial and residual culture should be the same.

A probable septic transfusion reaction was differentiated whenever associated clinical symptoms were present in conjunction with positive culture on the residual component, but recipient’s blood culture was not available or showed negative results. 

A possible septic transfusion reaction defined whenever the symptoms of post transfusion reaction existed in conjunction to recipient’s positive blood culture, but the residual culture was either negative or not available; however in this situation other causes for sepsis should be ruled out. The relevant data was gathered and processed (tables 1 and 2).

Any extra clinical symptoms, besides the underlying diseases, were observed and the outcomes of the patients were recorded as well.

The research protocol was approval by Ethics Committee of IBTO research center.

## Results

 Patients (aged 30 to 65 years; 65% male) underwent transfusion with random-donor PCs. The signs and symptoms of transfusion reaction by changes in the vital signs occurred in 12 (%0.4) cases; the average component transfused per patient was 4±2 PCs.

The study cases had acute lymphocytic leukemia (ALL), acute myeloid leukemia (AML), Hodgkin lymphoma, and non-Hodgkin lymphoma, after chemotherapy and irradiation therapy, but no history of post transplantation.

Almost all patients took at least one broad spectrum antibiotic, the 3rd or 4rd generation, namely cephalosporin, Vancomycin, Carbapenem or a combination of them.

None of the symptomatic patients were affected by other transfusion reactions.

Automated cultivation during 7 days of the incubation found 3 positive blood cultures. Among them one had heavy sinus infection and another had perineal abscess that caused bloodstream infection (Table 2); the cases were excluded from the study.

A 46-year-old male recipient was categorized as possible septic transfusion reaction, whose pulse rate and body temperature rose up from 80 to 85 bpm, and from 36.8ºC to about 38.4ºC, respectively; the patient was treated with broad spectrum antibiotics as shown in Table 2. The residue of the 3 transfused components (PCs), during the last 4 hours, was sent to IBTO microbiology laboratory, but no bacteria were detected. The sample of blood culture by automated cultivation method revealed the bloodstream infection caused by *Citrobacter Koseri*.

**Table 2:** Patients Suspected of Contamination with Platelet Transfusion

PC Culture Results	Patient’s Blood Culture Result	Consumption of Antibiotic	Disease	Vital Sign Before/After Platelet Transfusion				Age	Gender	Patient
				Body Temperature (°C)	Blood Pressure (mmHg)	Pulse recipient Rate	Rigors			
**Negative**	*Citrobacter* spp*.*	Yes	Leukemia	36.8/38.4	110/70 120/70	80/85	No/Yes	46	^Male^	1
**Negative**	Negative	Yes	Leukemia	38/38.5	110/80 100/80	78/84	No/Yes	52	^Female^	2
**Negative**	Negative	Yes	Lymphoma	37/38	110/70 110/70	80/82	No/Yes	25	^Male^	3
**Negative**	Negative	Yes	Leukemia	37.3/35	110/70 110/70	88/80	No/Yes	49	^Female^	4
**Negative**	Negative	Yes	Leukemia	37/37	130/80 130/80	85/80	No/Yes	49	^Female^	5
**Negative**	Negative	Yes	Leukemia	37/38.2	120/80 120/80	88/85	No/Yes	49	^Female^	6
**Unknown**	Negative	Yes	Leukemia	37.5/38	120/80 125/80	80/88	No/Yes	51	^Female^	7
**Unknown**	Negative	Yes	Leukemia	37/37	110/70 110/70	80/80	No/Yes	32	^Female^	8
**Negative**	Negative	Yes	Leukemia	38/37.8	100/80 100/80	84/88	No/Yes	70	^Male^	9
**Negative**	Negative	Yes	Leukemia	38.7/38.8	110/80 110/80	84/86	No/Yes	67	^Female^	10
**Unknown**	E coli	Yes	Leukemia	37/39.5	110/70 100/60	80/84	No/Yes	34	^Male^	11^*^
**Unknown**	E coli	Yes	Leukemia	37/38.5	120/80 125/80	82/82	No/Yes	52	^Female^	12 ♦

* Patient was excluded from the study due to perineal abscess as the possible source of bacteremia

♦ Patient was excluded from the study due to her sinus infection as the possible source of bacteremia

A 34-year-old male patient with leukemia transfused with 5 PCs showed an increase in pulse rate and body temperature from 80 to 84 bpm, and from 37ºC to 39.5ºC, respectively; *E. coli* was isolated from blood culture. He was excluded from the study due to perineal abscess, which could be defined as the source of bacteremia. Another patient was transfused with 4 PCs; a 52-year-old female and after that, her pulse rate and body temperature increased from 78 to 84 bpm, and from 38ºC to 38.5ºC, respectively; *E. coli* was isolated from blood culture. The recipient was also excluded from the study due to her sinus infection as a source of bloodstream infection.

## Discussion

Regarding vigorous achievements on controlling the viral transfusion-transmitted infections (TTI), now the bacterial contamination and associated septic reactions are the major infectious residual risks of transfusion. Bacterial contamination and its subsequent septic transfusion reactions are the second causes of mortality due to PCs transfusion. Bacterial contamination of blood components is the major infectious risk in transfusion medicine (11). The bacterial contamination rate of PCs depends on the production procedure and the employed microbiological culturing methods.

 This is the first report of septic transfusion reaction in Iran, based on the results of an active surveillance study held on the patients with hemato-oncological diseases as the most vulnerable and at risk group, due to their high exposure to PCs transfusion, in addition to immune-suppressed status.

Although the studies of screening by automated culture showed different results, the diversity in time of sampling, volume of inoculation, and culture conditions made their comparison hard (12-14). Even though the detected germs are predominantly originated from transient skin flora (11), gram negative organisms are mostly related to more severe septic reactions with high case fatality rate (2).

In the early 1990, bacterial contamination was the most common infectious complication arising after transfusion and caused about 14% to 24% of transfusion-associated mortality; but after implementation of preventive methods, the septic transfusion reaction decreased dramatically from 1:15 000 to 1:100 000 (12, 13, 15). Screening donors revealed interesting findings, such as occult colon adenocarcinoma, osteomyelitis, endocarditis, or pet snake bite, following the positive blood culture result (10).

The contamination rate of PCs are reported 1/2000 to 1/5000 in different blood centers with different strategies for microbial control, but it was also reported even as high as 1.06% (1, 15-18).

A mathematical modeling revealed that for each confirmed positive PCs, 19 collections might exist at low concentration of dormant bacteria, which could not be detected at the same time (19).

The current study reported a case with possible transfusion-transmitted septic reaction after random donor platelet transfusion, caused by *Citrobacter Koseri *contamination. It is an additional report of septic transfusion reaction caused by* Citrobacter Koseri*; previously, a case study in Brazil reported one case of septic transfusion reaction by the same bacteria in a 46-year-old female due to receiving 1 unit of packed red cell transfusion (10).

However, in a transfusion center in Ghana, *Citrobacter Frundei *was isolated from a blood component (11). Besides, in another study on a patient who received transfusion, there were references of *Citrobacter *spp. isolation (16).

*Citrobacter *species are present in the soil, water, food and in the gastrointestinal tract of mammalians. They behave as an opportunist microorganism, causing infection, mainly in patients with underling diseases.

Out of every 10 patients who received contaminated PCs, 4 cases presented septic shock (20). The clinical demonstrations of septic transfusion reaction could be unrecognised and underreported as a result of underlying disease or variability of signs, symptoms, and timing (21).

All patients received broad spectrum antibiotics before and during the transfusion that in fact could mask the symptoms of sepsis and produce false negative culture results. Most of the infectious complications occurred during 4 hours after transfusion; although some delayed bacterial complications may occur several days later.

No platelet contaminations were observed in the current study, which could be explained by insufficient volume of samples, additive solutions, sample conditions, the ability of apparatus to promote growth of bacteria, initial load of bacteria, etc. These conditions could present false negative results of the apparatus. Authors mentioned that the main reason might be inadequate volume of samples and the sample conditions, though it was insisted that this report was just a possible transfusion reaction, not definite or even probable; therefore, the possibility of nosocomial septic shock by sources other than blood products should be mentioned. The result of a blood cultivation study on 2285 patients admitted into a general hospital in Iran declared that more than 15% of positive blood cultures belonged to *Citrobacter* spp., and they mostly were resistant to many antibiotics, especially beta-lactams; thereafter, this opportunistic infection should be considered in hospital acquired infections. 

It could be assumed that PCs were contaminated with these opportunistic bacteria during processing or storage time. Microscopic defects might be formed during manufacturing of storage containers, or after manipulation during heat sealing, or the process of making sterile connection. Contamination of this type is most likely caused by environmental species (22).

Since 2006, IBTO implemented removal of an initial 10 to 40 mL sample of donated blood, which significantly improved the rate of bacterial contamination. Improved recommendations on blood donor medical deferrals policy with possible asymptomatic bacteremia reduced the risk, as well.

The hemovigilance and transfusion reaction issue is a new field of study. Appropriate clinical utilization of platelet transfusion and ongoing vigilance to recognize, investigate, promptly treat, and report all suspicious transfusion reactions are necessary to manage the patients with transfusion complications including TTBI. 

A potential limitation in the current study was the number of patients with lymphoma and leukemia. 

## Conclusion

Appropriate clinical utilization of PCs transfusion, and ongoing vigilance to recognize, investigate, promptly treat, and report all suspicious transfusion reactions are necessary to manage the patients /recipients with transfusion complication including TTBI.
